# Reactive granulomatous dermatitis arising in the setting of interleukin-17 inhibition successfully treated with tofacitinib

**DOI:** 10.1016/j.jdcr.2025.09.043

**Published:** 2025-10-14

**Authors:** Sarah L. Spaulding, A. Mitchel Wride, Michael J. Murphy, Muhammad H. Junejo, William Damsky

**Affiliations:** a Department of Dermatology, Yale School of Medicine, New Haven, Connecticut; b Department of Pathology, Yale School of Medicine, New Haven, Connecticut

**Keywords:** biologics, interleukin-17 inhibitors, interstitial granulomatous dermatitis, interstitial granulomatous drug reaction, reactive granulomatous dermatitis

## Introduction

Reactive granulomatous dermatitis (RGD) encompasses a spectrum of cutaneous granulomatous disorders. Classically, these eruptions had been divided into interstitial granulomatous dermatitis, interstitial granulomatous drug reaction, and palisaded neutrophilic and granulomatous dermatitis. However, more recently RGD, which encompasses these three diagnoses which may overlap with one another, has become the preferred nomenclature. RGD is most commonly associated with inflammatory arthritis and systemic lupus erythematosus, as well as the use of certain medications, including tumor necrosis factor inhibitors.^1^^,^^2^ Here we report a case of longstanding RGD that arose in the setting of interleukin (IL)-17 inhibition with secukinumab for psoriasis and persisted for years until successful treatment with tofacitinib.

## Case report

**Fig 1:**
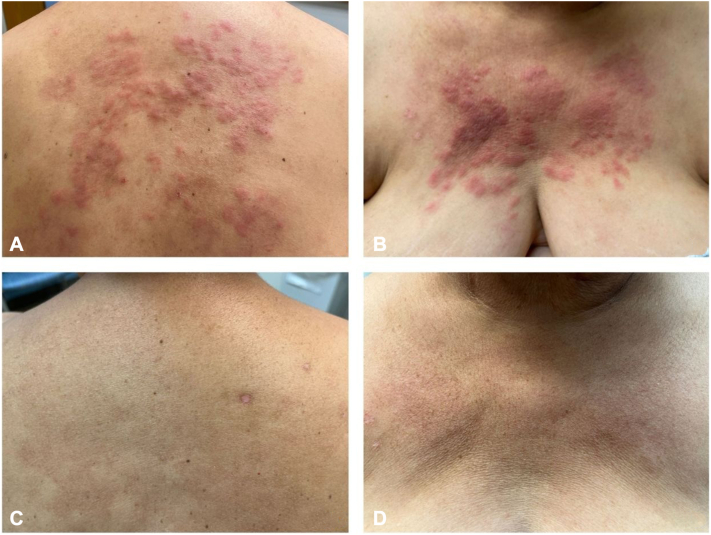
**A** and **B,** Diffuse erythematous and slightly edematous papules and focally annular plaques before tofacitinib. **C** and **D,** Clearance of the eruption is noted 2 months after initiation of tofacitinib.

A 61-year-old woman presented to our clinic with pruritic skin lesions on her upper back, chest, and bilateral arms (Fig 1, *A* and *B*). She had a history of psoriasis and psoriatic arthritis, well controlled on adalimumab 40 mg and methotrexate 20 mg weekly for 2 years until she developed a pleural effusion, which led to discontinuation of adalimumab. Methotrexate was also discontinued due to elevated liver function tests. Subsequent exacerbation of her psoriasis prompted initiation of secukinumab 150 mg monthly, increased to 300 mg monthly after 4 months. In the fifth month of secukinumab treatment, she reported development of a widespread rash on her chest, back, and arms. She was transitioned from secukinumab to risankizumab, with sustained control of her psoriasis, yet no improvement of the new rash. Apremilast was trialed in place of the risankizumab 6 months later but discontinued after 3 months due to lack of response. Intermittent intramuscular injections of triamcinolone resulted in temporary, mild improvement of the new rash. Risankizumab was restarted, and she had continued this therapy until her initial evaluation in our clinic, 3 years after the new rash onset.

**Fig 2:**
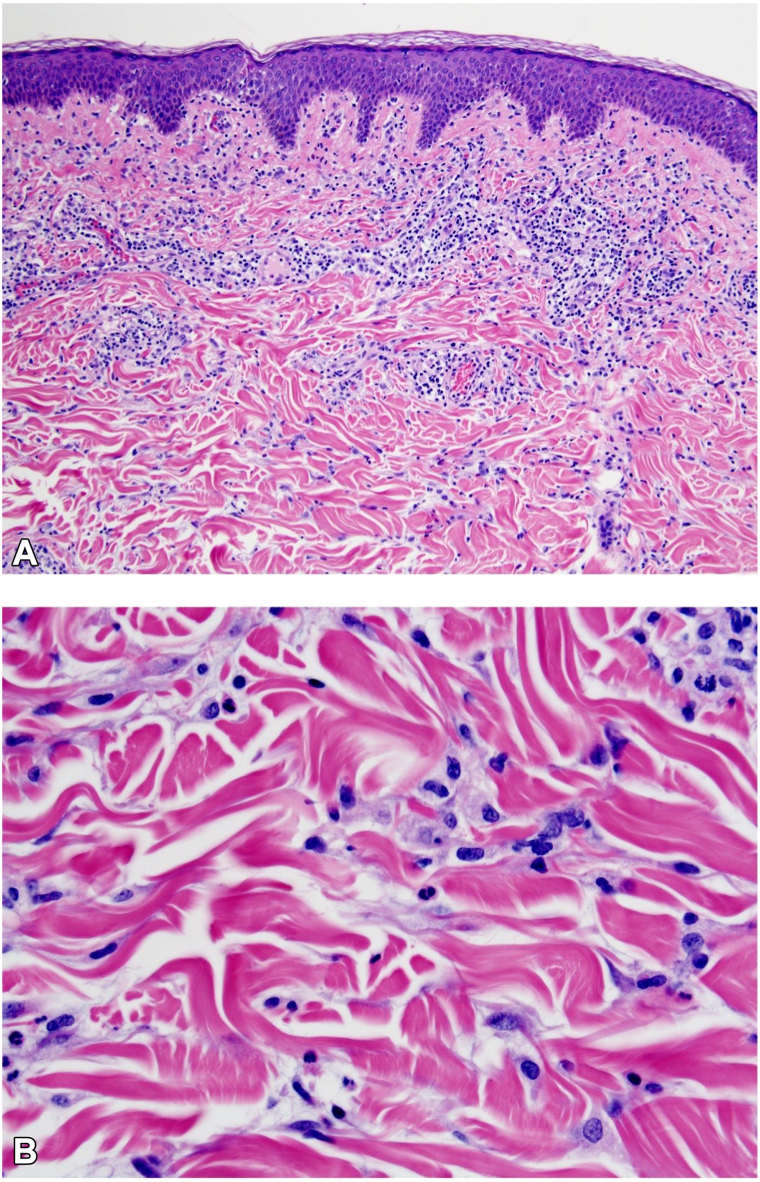
Histopathology. **A**, Focal vacuolar change along the dermoepidermal junction overlies interstitial granulomatous inflammation, including interstitial histiocytes. **B,** Higher power view showing interstitial histiocytes without significant collagen alteration or mucin. Hematoxylin & eosin stain; magnification 100× (left), 400× (right).

**Fig 3:**
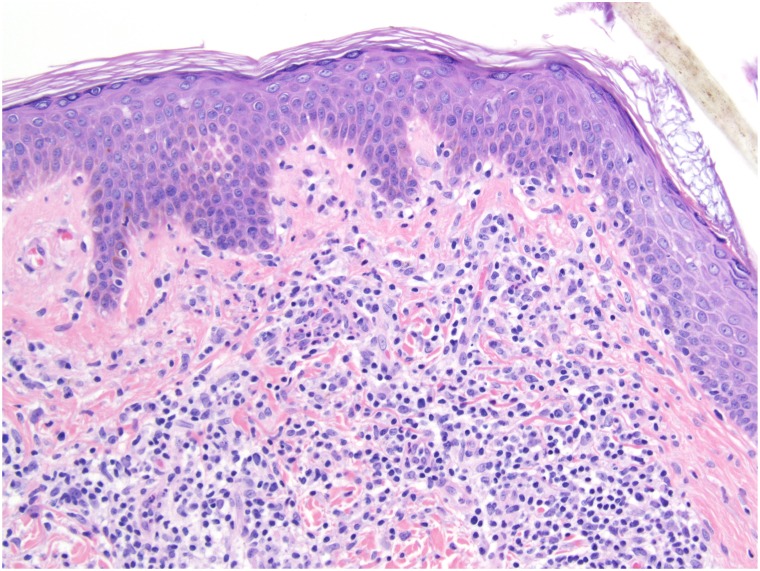
Vacuolar change along the dermoepidermal junction, higher power view.

The patient’s medical history was notable for IgG lambda monoclonal gammopathy of unknown significance (MGUS) that was deemed by her hematologist to not require treatment or bone marrow biopsy due to normal complete blood count, as well as type 2 diabetes mellitus, hypertension, and hyperlipidemia. The patient’s other medications included amlodipine, which had been initiated after the new rash appeared. Review of systems was unremarkable. Skin exam revealed erythematous papules and focally annular plaques on a background of light pink-brown patches on the upper chest, back, and bilateral arms (Fig 1, *A* and *B*). Two separate skin biopsies both revealed vacuolar interface change and interstitial granulomatous inflammation without significant collagen alteration or mucin (Figs 2 and 3). Special stains for micro-organisms were negative and neutrophils were largely absent. Based on the clinicopathological findings, a diagnosis of RGD was favored. Antinuclear antibody test was negative.

To evaluate the potential role for interferon gamma (IFN-γ) in this patient’s RGD, we performed *IFNG* RNA in situ hybridization staining on the skin biopsy.^3^ This showed 2-3+ staining in the areas of granulomatous inflammation, comparable to the levels of staining seen in sarcoidosis and granuloma annulare (GA). *IFNG* mRNA staining is not observed in normal skin in the authors' experience.^3^

Due to the longstanding presence and severity of her RGD, combined with the molecular data implicating IFN-γ, the patient was started on tofacitinib 5 mg twice daily. Tofacitinib resulted in complete clearing of the RGD within 2 months (Fig 1, *C* and *D*). Attempts to taper and then discontinue the tofacitinib have led to recurrence of the RGD. Her psoriasis was not well-controlled on the tofacitinib and concomitant risankizumab was initiated. She has remained stable of this regimen for over 1 year.

## Discussion

IL-17 inhibitors have been associated with development or unmasking of sarcoidosis and GA.^4^ Crohn’s disease, which may also show granulomatous inflammation, may also develop or worsen in the setting of IL-17 inhibition. We are unaware of reports of RGD in the setting of IL-17 inhibition. It is thought that IL-17 inhibition may promote granulomatous inflammation via immune polarization switch from Th17 towards Th1 (and increased IFN-γ) and possibly other mechanisms.

Most likely, the IL-17 inhibition in this patient unmasked a predisposition (underlying psoriasis, psoriatic arthritis, or MGUS) to develop RGD. GA was considered in the differential diagnosis of this patient but not favored due to the lesion distribution, lesion morphology (bright, not dull, pink), presence of vacuolar interface dermatitis on biopsy, and lack of significant collagen alteration or mucin on biopsy. It is also possible that this cutaneous granulomatous inflammation was related to the patient’s underlying MGUS, as RGD has previously been associated with gammopathy.^1^ The timing of the rash, having appeared shortly after initiation of secukinumab, suggests that the drug is the more likely proximal driver of the RGD in this case. Further, tofacitinib is generally not felt to treat MGUS. Monitoring of the MGUS in this patient over time will be important.

IFN-γ is considered to be an important driver of conventional inflammatory granulomatous skin diseases such as sarcoidosis and GA.^5^ Tofacitinib has been shown to be effective in these other inflammatory granulomatous diseases and is thought to work primarily through inhibition of IFN-γ.^5^, ^6^, ^7^, ^8^, ^9^ Tofacitinib inhibits IFN-γ by blocking downstream Janus kinase 1/2, as well as other cytokines. We hypothesize that a similar mechanism may be involved here. Tofacitinib was selected given its more potent inhibition of IFN-γ compared to deucravacitinib, which might have been effective for her underlying psoriasis. Overall, this case suggests that IL-17 inhibition may be associated with IGD and demonstrates the efficacy of Janus kinase inhibition in treating longstanding and/or severe cases.

## Conflicts of interest

Dr Damsky is a consultant for Pfizer, Incyte, Eli Lilly, and TWi Biotechnology, has research funding from Pfizer, Advanced Cell Diagnostics/Bio-techne, and AbbVie and receives licensing fees from EMD/Millipore/Sigma, all outside the submitted work. Dr Wride was supported by the National Institute of Diabetes and Digestive and Kidney Diseases of the National Institutes of Health under award number T35DK104689. Drs Spaulding, Murphy, and Junejo have no conflicts of interest to disclose.
